# Development and validation of a measurement instrument for physical activity-related health literacy (PA-HL): a study protocol

**DOI:** 10.1186/s13690-025-01661-w

**Published:** 2025-06-23

**Authors:** Elena Fitzner, Thomas Hering, Kevin Dadaczynski

**Affiliations:** 1 Faculty of Human Science, Department of Sport and Health, University of Potsdam, Karl-Liebknecht-Straße 24-25, Potsdam, 14476 Germany; 2 Research Department Health Industries & Quality of Life, Westphalian University of Applied Sciences - Institute for Work and Technology, Munscheidstraße 14, 45886 Gelsenkirchen, Germany; 3 Department of Applied Human Sciences, University of Applied Sciences Magdeburg-Stendal, Osterburger Straße 25, 39576 Stendal, Germany; 4 Centre for Applied Health Science, Leuphana University Lüneburg, Wilschenbrucher Weg 84a, Lüneburg, 21335 Germany

**Keywords:** Study protocol, Physical activity, Health literacy, Measurement instrument development, PA-HL

## Abstract

**Background:**

Physical activity (PA) constitutes an effective strategy for the prevention and management of non-communicable diseases (NCDs). Research demonstrates that a substantial proportion of the adult population in Germany does not reach the recommendations for PA. Health literacy (HL) is significantly associated with health behaviours, including PA levels, and various health outcomes. However, no measurement instrument currently exists that assesses HL within the PA context whilst focusing on information processing competences. Therefore, this research aims to integrate HL and PA concepts and to develop a novel measurement instrument for physical activity-related HL (PA-HL) in Germany emphasising information processing competences.

**Methods:**

The development and evaluation process employs a mixed-methods design. The instrument development follows a three-phase methodology: First, the conceptualization of PA-HL is defined, including its dimensions and information processing competences with PA focus. Second, during scale development, content and face validity of items are tested through a three-round eDelphi process with experts in HL and PA fields, and cognitive interviews with adults. Third, the novel instrument undergoes psychometric testing in a cross-sectional pilot study utilising principal component analysis and confirmatory factor analysis.

**Discussion:**

Through utilising health information on PA, individuals should be enabled to influence their own PA behaviour and that of others to maintain and promote health. The novel PA-HL measurement instrument could be employed in future research and practice to identify the population support needs and difficulties in processing health information on PA. This should facilitate the development and evaluation of public health interventions tailored to the specific needs of population group requirements.

**Table:** 

Text box 1. Contributions to the literature
• From a public health perspective, physical activity represents one of the most significant approaches to health care, disease prevention and health promotion. Therefore, examining competences to deal with physical activity-related health information is of particular relevance to public health science, practice and policy.
• The development of the novel measurement instrument presented herein will advance health literacy research within the physical activity domain and contribute to the growing body of domain-specific health literacy literature.
• This novel physical activity-related health literacy measurement instrument will enable future identification of suboptimal information processing competences regarding physical activity across diverse population groups, thereby facilitating the development and evaluation of targeted public health interventions

## Background

Physical activity (PA) (among other factors) plays an important role in decisions to maintain and promote health. PA is particularly important for preventing diseases and maintaining quality of life [1], and is defined as any movement of the body that results in energy expenditure [2]. PA encompasses occupational, sporting, domestic and other activities, with exercise and sporting activities being a subset of PA [3]. PA is an effective primary and secondary preventive intervention strategy for at least 25 non-communicable diseases (NCDs), as regular PA has a positive effect on the cardiovascular system [4], musculoskeletal health [5, 6], cognitive abilities [7] and mental health [8]. Physical inactivity, on the other hand, significantly increases the risk of prevalence and mortality from NCDs [9]. Physically active people have an overall lower risk of cardiovascular mortality, breast and prostate cancer, bone fractures, disabilities in everyday life, dementia, depression and an overall higher quality of life, experience a healthier ageing process [10] and have a lower risk of premature death from heart disease and strokes [11]. However, these empirical findings are contrasted by the PA behaviour of the population. The global prevalence of physical inactivity was 28% in 2016, with prevalence being particularly high in high-income countries and rising continuously since 2001 [12]. In Germany, the level of low PA is 21.1% of persons aged ≥ 15 years (95%CI: 18.2—24.1%), which is lower than in other European countries but still represents a high number of inactive people [13].

Barriers to PA and interest in PA are partly driven by personal or behavioural aspects, with younger adults (< 50 years) reporting lack of time as a reason for inactivity and older adults mentioning little enjoyment of PA and health problems as barriers [14]. This raises the question of whether health information on PA sufficiently addresses these aspects of time management, enjoyment and health benefits to meet the needs of the population, or whether there are further barriers in dealing with health information on PA. The underlying factors for information processing difficulties related to health literacy (HL) and PA are inadequately characterised.

The concept of HL has gained prominence in public health research, policy and practice and is defined by Sørensen et al. [15] as:"… people's knowledge, motivation and competences to access, understand, appraise and apply health information in order to make judgements and take decisions in everyday life concerning health care, disease prevention and health promotion to maintain or improve quality of life during the life course."([15], p.3). Accordingly, HL is understood as a general information processing competence that encompasses various health-related areas and aims to maintain and improve the health of the population while enabling individuals to make informed decisions and take control of their health-related actions [16].

Many systematic reviews and meta-analyses show positive associations between HL and PA [17–20]. However, an intervention study to increase PA showed that HL had no moderating effect on the outcomes of participants'activity behaviour. The intervention was successful regardless of the participants'HL level [21]. A limitation of such analyses is that HL assessments frequently employ general and very broad measurement instruments without specific consideration of PA contexts. Consequently, the current scientific evidence remains insufficient and demonstrates the need for a measurement instrument capable of providing precise insights into the relationship between PA-related health information processing and PA behaviour. To date, it is unclear how to measure the competences required to deal with PA-related health information (and possibly influence PA levels). There are concepts and measurement instruments in this area, such as the physical activity-related health competence model and questionnaire (PAHCO_12) [22] or the concept of physical literacy [23]. The approach of Carl et al. [22] focuses on 1) movement competences, including basic motor skills and abilities, 2) control competences, including basic body and movement-related knowledge, and 3) movement-related self-regulation competences, including characteristics such as self-efficacy and motivational structures. Physical literacy is based on three elements, namely 1) motivation and confidence (affective), 2) physical competence (physical), and 3) knowledge and understanding (cognitive) that lead to lifelong engagement in physical activities (behavioural) [23]. In contrast to the HL approach of Sørensen et al. [15], both concepts do not focus exclusively on information processing competences. The four core dimensions of health information processing—access, understanding, evaluation and application—as conceptualised by Sørensen et al. [15], remain absent from these conceptualisations and corresponding measurement instruments. Oppermann [24] describes the similarities and differences between HL and physical activity-related health competence: both concepts have a different theoretical basis and are characterised by a different understanding of HL. The difference between the PAHCO model [22] and the HL model by Sørensen et al. [15] lies in the specific focus and conceptual orientation: PAHCO primarily considers the physical, motivational and volitional aspects of exercise and aims to promote physical activity by addressing the following key dimensions: motor skills (e.g. being able to lift an object of about 25 kg), control of physical effort (e.g. knowing how to use physical training to improve endurance), body awareness (e.g. sense of posture), emotional attitudes (e.g. emotions at the thought of physical activity) and others. This model does not include information processing—with the associated four competences of accessing, understanding, evaluating and applying health information. Sørensen's model also emphasises the empowerment aspect, but in contrast focuses on the ability to make health-related decisions for oneself and others across the lifespan in different areas, including disease management, prevention and health promotion. The focus here is on health information, e.g. to find out where to get professional help, to assess the benefits of treatment options and to make decisions to improve health. Nevertheless, both approaches can benefit from each other [24]. Individual facilitators and barriers to PA adoption as health-promoting behaviour require further elucidation. Particular attention is given to health information processing within PA contexts, as this constellation remains insufficiently addressed in the above-mentioned concepts. They either focus on health information on PA AND other topics such as nutrition in the broader context of a healthy lifestyle or on PA such as motor skills, knowledge, motivation WITHOUT considering PA-related information processing competences.

The sheer volume of health information available raises the question of how to deal with this information. Adults with PA-related health information behaviours who watched health-related YouTube videos were more likely to achieve the levels of physical activity recommended in clinical practice guidelines [25]. However, there appears to be no relationship between barriers to physical activity and physical activity information behaviour [26]. In these studies on information-seeking behaviour, no other health information processing competences besides information seeking (understanding, evaluating and applying) are considered. Therefore, the focus of this project is on all four core dimensions of health information processing.

### Study aims and objectives

There is no measurement instrument that specifically addresses physical activity-related HL (PA-HL) focusing on information processing competences as defined by Sørensen et al. [15]. Therefore, the primary aim is to develop and validate a measurement instrument for PA-HL. The aim is to investigate information processing competences for PA-related information in Germany. The following study protocol describes the methodology of the development process considering the following research questions:

1. How can the concept of HL, based on the conceptualisation by Sørensen et al. [15], be applied to the topic of PA?
2. Which dimensions and items prove to be relevant for the operationalisation of PA-HL?
3. What is the validity and reliability of the new PA-HL measurement instrument?

## Methods

This research is based on a mixed-methods design and will be conducted in Germany from May 2023 to November 2026.

### Design of the study

**Fig. 1:**
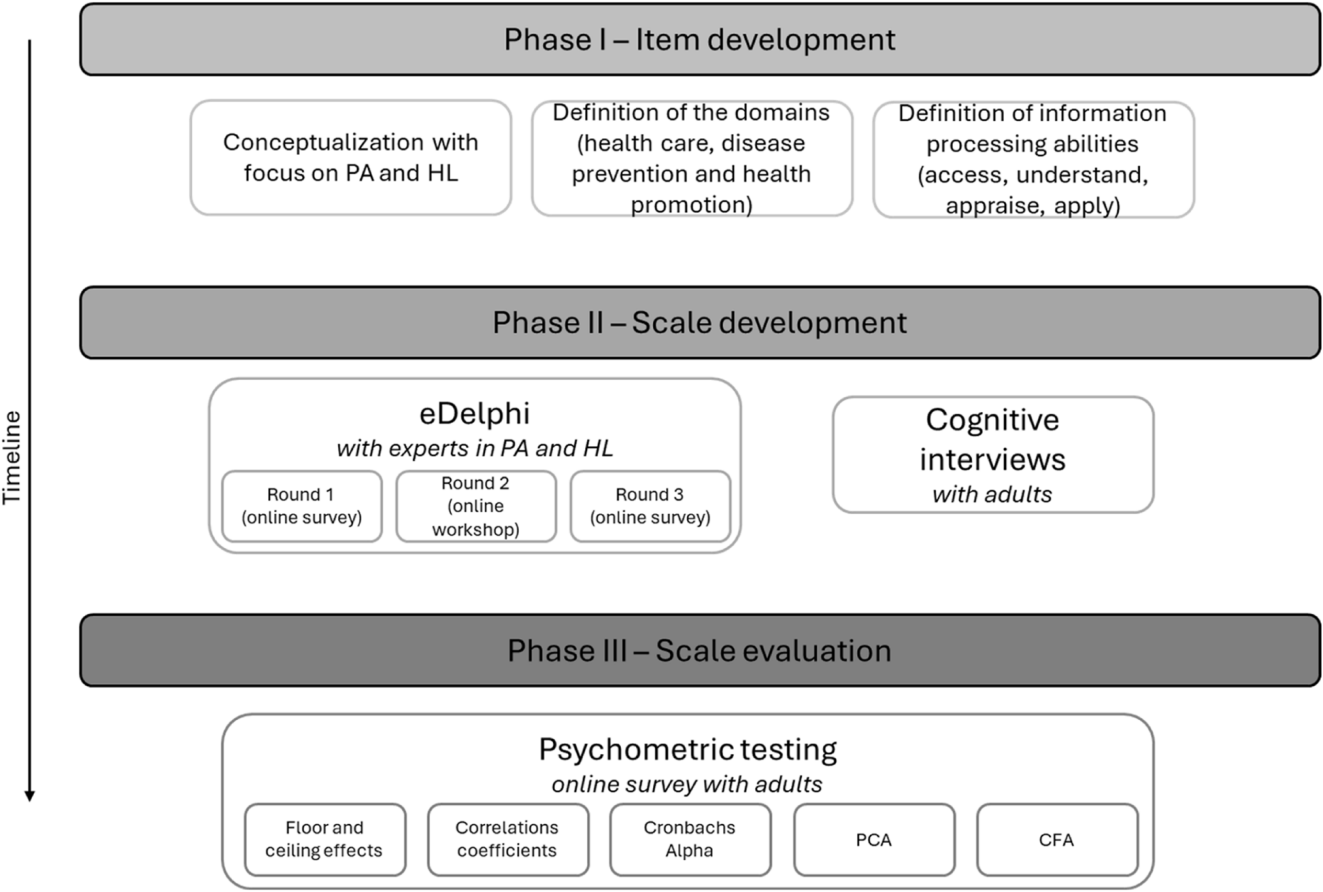
Study design for a three-phase development process for the measurement instrument on physical activity-related health literacy among German adults, 2023—2026

Following the guidelines of Boateng et al. [27], this research consists of three phases (see Fig. 1).

#### Phase 1: literature search and item generation

The first step is to define the concept and domains of the measurement instrument, focusing on the areas of health care, disease prevention and health promotion. The HL conceptualisation by Sørensen et al. [15] serves as a conceptual framework. The definitions of the competences for accessing, understanding, appraising, and applying information are based on Bitzer & Sørensen [28]. Using a deductive item generation method, a preliminary item pool is developed based on an in-depth analysis of existing HL instruments and a literature review (in Medline and PubMed on predictors of PA and information-seeking behaviour for PA). Existing measurement instruments are used to delimit the content of the measurement instrument in order to avoid overlaps. As no comparable instruments on PA exist to date, general HL measurement instruments are identified and items are transferred to the topic of PA. According to the widely accepted conceptual understanding of Sørensen et al. [15], PA-HL is (preliminary) defined as:"PA-HL is linked to literacy and entails people's knowledge, motivation and ability to access, understand, appraise and apply relevant health information about physical activity in different forms in order to make everyday decisions to incorporate physical activity for disease management, disease prevention and health promotion in order to maintain or improve quality of life."Exemplary items developed during the initial phase, grounded in generic HL dimensional framework, are presented in Table 1.

**Table 1:** Health literacy matrix on exemplary items in the three dimensions and four competences of physical activity-related health literacy

Dimension/Competence	Access/obtain health information about physical activity	Understand health information about physical activity	Process/appraise health information about physical activity	Apply/use health information about physical activity
	On a scale from very easy to very difficult, how easy do you think it is to …			
Health care	… find information about how physical activity can relieve illness and physical discomfort?	… understand information that doctors or other professionals (e.g. physiotherapists or sports therapists) recommend to you about physical exercise for illness and physical discomfort?	… judge whether information (e.g. in magazines, advertisements, on the Internet, …) about physical exercise for illnesses and complaints (e.g. back training, walking, …) is trustworthy?	… use physical exercise to treat diseases and ailments based on the information you found?
Disease prevention	… find information about whether physical activity prevents disease?	… understand from information (e.g. in magazines, adverts, on the internet) whether physical activity helps to prevent illness?	… judge how much physical activity you should do to prevent illness?	… decide, based on recommendations, how long and how intensively you should be physically active to prevent the onset of a disease?
Health promotion	… find information about how physical activity can improve your health?	… understand information about signs of high physical exertion (e.g. fatigue, shivering, increased heart rate)?	… assess how the amount and intensity of your physical activity affects your physical and mental health?	… be sufficiently physically active in your everyday life to improve your health based on the information you have found?

#### Phase 2: scale development (conversion of individual items into a measurement instrument)

Secondly, a three-stage eDelphi process will be conducted to assess the preliminary item pool and to test content validity, following the Guidance on Conducting and REporting DElphi Studies (CREDES) [29]. An eDelphi is a method that can be used as an evaluation and assessment tool to reach consensus on research and development issues, e.g. on the relevance and comprehensibility of items [30]. The eDelphi encompasses three iterative rounds, preceded by pretesting with independent reviewers who had no prior involvement in item development or the subsequent eDelphi procedure. In the first and last round, an online survey is conducted in which the experts are asked to rate each item with regard to the indicators of content validity: (1) relevance to the definitions of the individual items and presentation of the respective definition and (2) clarity and comprehensibility of the item [31]. A 4-point Likert scale is used to assess relevance (very relevant, relevant, moderately relevant, irrelevant) and comprehensibility (very simple, simple, difficult, very difficult). In the second eDelphi round, an online workshop is held with experts to discuss the structure and particularly heterogeneous results of the first round. Between the eDelphi rounds, the feedback is summarised and sent to the participants in anonymised form [32]. Based on the results of the eDelphi rounds, the item pool is reduced, adapted or expanded.

In addition, in this second phase, cognitive interviews are conducted with potential end-users to assess whether the item pool is appropriate for the objectives of the measurement instrument, to assess face validity [27] and to further develop and test the instrument [33]. Cognitive interviews have proven to be a relevant test procedure in the development and evaluation of measurement instruments [34]. These interviews are used to introduce the new measurement instrument to the target group and to identify potentially problematic items or difficulties in answering the questions [33] as well as underlying causes for the adaptation of the instrument [34]. A heterogeneous sample will be interviewed to analyse how adults understand PA-HL items and to identify potentially problematic items and difficulties in answering the questions [33, 35]. For this purpose, the methods of thinking aloud and probing are used [33].

#### Phase 3: scale evaluation

In the third step, an online survey is conducted to evaluate the novel measurement instrument and assess its psychometric quality. The online survey is conducted after a pretest to check the technical functionality, comprehensibility and logic of the questionnaire. The detailed description of the statistical calculation is described in the statistical analysis section.

### Sample, sample size and recruitment strategy

#### eDelphi

The participants for the eDelphi are contacted by email. For the Delphi method, Niederberger & Renn [30] describe that the number of experts involved depends on the number of items or hypotheses and the expected response rate, but do not give any recommendations for the minimum number of cases required. The study will include a minimum of 12 participants. The participants of the eDelphi study are experts in the fields of HL, PA, physiotherapy or public health. Professional experience and expertise in one or more of the aforementioned research areas serve as inclusion criteria.

#### Cognitive interviews

For the cognitive interviews, Pohontsch & Meyer [33] recommend between five and 15 interview partners. This means that this study will include at least five adults over the age of 18 years. As the measurement instrument is to be used in different settings (health care, prevention, health promotion), participants from different age groups and with different educational, cultural and religious backgrounds are included. Participants are recruited for the cognitive interviews using the snowball method. Personal contacts are asked about their connections to people of different age groups (e.g. students to pensioners), different occupational fields (e.g. crafts, retail, secretarial work) as well as to unemployed people and people with different cultural backgrounds. These connections are used to establish contact with potential dialogue partners.

#### Scale evaluation (Pilot study)

The scale will be evaluated in a quantitative cross-sectional pilot study with a convenience sample. Adults aged 18 years and older will be recruited. The estimated sample size is based on Boateng et al. [27] and requires 10 respondents per survey item or 200 to 300 respondents per observation [36, 37]. In this study, the aim is to include at least 200 respondents, as the final number of items has not yet been determined. The recruitment strategy includes various channels such as personal requests to community-based centres, organisations and associations (e.g. local NGOs or cultural centres), social media, institutional partnerships and personal contacts to reach a suitable sample. Access is possible via a link or QR code that is distributed via email and social media and can be printed out and posted in the local organisations or centres.

### Variables

In the eDelphi method, socio demographic characteristics (e.g. gender, age, employee status, professional background, work experience) are collected in addition to the validity indicators. In the cognitive interviews, sociodemographic characteristics are collected to understand the composition of the sample and to contextualise the results (i.e. gender, age, education level, income, place of residence, chronic diseases, migration background) as well as the participants'understanding of the PA-HL construct and their interpretation of the items and misconceptions.

As comparative characteristics are required to assess construct validity in the scale evaluation phase, further standardised measurement instruments and knowledge-related items are included in the scale evaluation. The measurement instruments under consideration for construct validity assessment are presented in Table 2 (section comparative measurement instruments).

**Table 2:** Overview of the variables and measurement instruments employed in the pilot testing phase for the physical activity-related health literacy scale evaluation, 2025—2026

	Measurement instrument	Content	No. of items	Psychometric information
Socio demo-graphic characteristics	none	gender, age, level of education, income, place of residence, chronic diseases, migration background	7	none
Physical Activity-related Health Literacy	Physical Activity-related Health Literacy Questionnaire PA-HL-Q	competences for finding, understanding, assessing and applying health information relevant to physical activity	In development	to be tested
Comparative measurement instruments	European Health Literacy Questionnaire HLS-EU-Q12 [38]	General health literacy	12	Cronbach Alpha 0.67 to 0.87; good data model fit through factor confirmatory model; content and face validity is ensured by using theory-based matrix. Health literacy Survey (2019) questionnaire short form (HLS-19-Q12) score correlates sufficiently highly (*r* ≥ 0.897) with equivalent score for the Health literacy Survey (2019) questionnaire long form (HLS19-Q47) [38]
	International Physical Activity Questionnaire – Short Form IPAQ-SF [39]	Physical activity in the last 7 days	9	Strong positive relationships between activity monitor data and IPAQ data for total physical activity (*r* = 0.55, *p* < 0.001) and vigorous physical activity (*r* = 0.71, *p* < 0.001) [40], acceptable validity in assessing levels of physical activity [41]
	Health Action Process Approach HAPA [42, 43]	describe, explain, and predict changes in health behaviors in a variety of settings	65	evaluated with an acceptable content validity, model fit and Cronbachs Alpha in a related context [44]
	Knowledge-related items according to the method of van der Vaart & Drossaert [45]	measure more than people’s perceived literacy skills with performance-based items	7	Not applicable Context in this study: 1) trustworthy sources of information, 2) knowledge about current guidelines, 3) knowledge about the content of exercise recommendations

### Statistical analysis

All statistical analyses are carried out using IBM SPSS Statistics, V.24.0 (IBM), and the statistical software R, V.3.5.2, for quantitative analysis. Descriptive statistics are calculated for the socio-demographic data.

#### eDelphi

The Item-Level Content Validity Index (I-CVI) and the average Scale-Level Content Validity Index (S-CVI/Ave) are calculated for the eDelphi^1^ [46, 48]. The I-CVI measures the agreement between the expert judgements for an individual item with regard to the relevance of the item and the S-CVI determines the content validity of the entire measurement instrument. The I-CVI should not be lower than 0.78 for the inclusion of an item [48] and an S-CVI/Ave of 0.90 or higher is acceptable [49]. High values therefore indicate that the items/scale were assessed as valid in terms of content.

#### Cognitive interviews

The cognitive interviews are analysed descriptively and content-analytically [50]. For this purpose, all audio files are transcribed using key points [51]. Following Prüfer & Rexroth [35], all statements made by the interviewees for each item are compiled in a document that provides separate analysis for each item. The statements are assigned to the"Questionnaire Appraisal Coding Scheme"[52] and analysed [35]. Items will be linguistically modified or eliminated based upon consensual feedback from the results. Particularly inconsistent results and their implications for the item pool are discussed in a group of five researchers in order to reach a consensus on how to deal with these ambiguous responses.

#### Scale evaluation (Pilot study)

Psychometric testing is one of the most important steps in the development of a measurement instrument [53, 54].

Floor and ceiling effects are analysed by calculating percentages for the lowest and highest possible scores. The established threshold for determining a floor or ceiling effect is 15% [54]. The content and face validity of the measurement instrument is checked and improved by conducting the eDelphi procedure and cognitive interviews.

Spearman's rank correlation coefficient (rs) is used to analyse construct validity and interpreted as follows: 0 to 0.25 little or no association; 0.25 to 0.50 adequate association; 0.50 to 0.75 moderate to good association and above 0.75 good to excellent association [55].

To validate the novel measurement instrument, both principal component analysis (PCA) and confirmatory factor analysis (CFA) are used. A split-sample PCA and CFA approach is used to analyse multidimensionality. For this purpose, the sample is randomly divided into two groups so that one group is used for PCA and the other for CFA [37, 56]. This statistical approach was selected as CFA relies upon theoretical foundations that is preferable for confirmatory hypothesis testing. PCA is utilised to reduce the number of original items in the planned instrument to a smaller number.

For PCA, a screen plot is used to determine the suitability of the data set for data reduction and the number of statistically meaningful dimensions [57]. An orthogonal rotation is performed so that the items load as high or low as possible on a factor and show the final and interpretable loading structure [58]. Items that do not load sufficiently on a factor (< 0.30) are excluded.

CFA is a procedure for testing relationships between observable variables and latent variables [59] and is therefore regarded as a central test instrument for measurement models for hypothesised constructs [60]. In this case, a theoretical model is developed a priori based on the results of the eDelphi procedure and the model proposed by the PCA to be tested. A variance–covariance matrix is calculated and used to estimate the model parameters [60]. In addition, the factor loadings are calculated, which represent estimates of the correlations between the item variables and the latent variable. The higher the factor loading, the greater the proportion of the measurement variance that is explained by the factor [37, 56, 59]. The quality of the CFA is assessed at both the construct and the model level. The construct quality is analysed using the following indicators: factor loadings (significant, > 0.5 [61]), factor reliability (> 0.6 [62]) and average variance extracted (AVE > 0.5 [63]). The overall quality of the model is assessed using the following indicators: X2 (not significant), χ2/df (< 2–3), GFI (> 0.95), CFI (> 0.90), SRMR (< 0.1), RMSEA (< 0.05) and the 95% confidence interval for RMSEA = [0; 0.05] [61].

Cronbach's alpha is used as a measure of reliability [64] and a value of 0.65 to 0.80 is considered appropriate [65, 66]. The minimum value for clinical use is α = 0.90 [54].

### Ethics and dissemination

The study was designed according to the principles of the Declaration of Helsinki. The study received ethical approval from the Ethics Committee of Fulda University of Applied Sciences on 21 December 2023, with an amendment for cognitive interviews approved on 21 June 2024 (reference EK231204).

## Discussion

It has been shown that HL is closely related to health behaviour [67] and that higher levels of HL enable people to make better decisions about their PA [17]. Health behaviour and HL constitute broad fields encompassing numerous domains (e.g. diet, smoking, vaccination). Therefore, to provide a meaningful starting point for promoting physically active lifestyles in the future, it is essential to assess information processing competences on a PA-specific basis. This protocol outlines the development of a novel measurement instrument and establishes the rationale for a PA-HL measurement instrument grounded in Sørensen et al.'s [15] information processing framework, constituting a systematic approach to HL instrument development and validation within PA contexts. By focussing on information processing competences, it closes the gap to previously established measurement instruments that are generic or based on concepts other than the information processing approach of Sørensen et al. [15]. The approach presented here focuses on health information about PA and the competences people need to deal with PA-related health information. In contrast to existing generic HL measurement instruments, it will be possible to investigate relationships between PA-related information processing and PA-related behaviour in more detail, which in turn will allow for more precise recommendations for tailored support.

Once the measurement instrument is developed and psychometrically tested, it could be utilised in research and practice, e.g. in future HL studies or in physiotherapy settings, rehabilitation programmes, or health promotion and prevention interventions to gain a better understanding of individuals'competences. With the results of this PA-HL measurement instrument, healthcare providers, researchers or authorities will be able to provide targeted information and efficiently support their clients and populations in the information process. The newly introduced construct PA-HL is the first that aims at a direct conceptual link between PA and HL by taking information processing into account.

In summary, there is a great need to understand how information processing competences related to PA predict activity-related behaviour and thus various health outcomes such as NCDs.

### Limitations

There are some limitations regarding the methodology, conceptualization and validation. As with general HL, it must be considered that individual competences are influenced by enabling factors external to the individual, such as access to information and support in critical appraisal. Such enabling factors and contextual conditions are often lacking in individual HL [68]. One concern is that using the I-CVI and summarising the experts'ordinal multi-point ratings into two categories (relevant/not relevant) may lead to a loss of information and that the I-CVI does not capture whether a measurement instrument contains a comprehensive set of items to adequately measure the construct of interest [46]. To address this limitation, open-ended feedback will be analysed in the first and third eDelphi rounds. The scale evaluation is conducted using a random sample, which could lead to selection bias. Nevertheless, the aim is to achieve as balanced a sample as possible in terms of socio-demographic characteristics. Therefore, different distribution channels are utilised to reach diverse population groups and minimise this bias [69]. Future results will be analysed accordingly, taking into account the socio-demographic distribution, and subgroup analyses will be conducted to investigate differences based on socio-demographic characteristics. In addition, access to the survey will be kept as simple as possible to avoid drop-outs due to technical barriers.

### Implications

This measurement instrument could be used by experts in research and practice as a screening tool. In addition, the results of this PA-HL measurement instrument might reveal the needs and challenges of individuals in information processing so that experts can provide tailored-based support and specific PA-related health information in the future. From a public health perspective, the measurement instrument can be used in future studies to gather information on information processing competences of PA in the population and to develop future interventions with communities or authorities. In general, this study supports research in the field of HL and PA and raises awareness of both topics in relation to health care, prevention and health promotion.

## Data Availability

No datasets were generated or analysed during the current study.

## Abbreviations

CFA: Confirmatory Factor Analysis
CREDES: Guidance on Conducting and Reporting Delphi Studies
HL: Health Literacy
HLS-19-Q12: Health literacy Survey (2019) questionnaire short form
HLS-19-Q47: Health literacy Survey (2019) questionnaire long form
I-CVI: Item- level Content Validity Index
NCD: Non-communicable diseases
PA: Physical Activity
PA-HL: Physical activity-related health literacy
PCA: Principal Component Analysis
S-CVI(Ave): Average Scale-level Content Validity Index

## Footnotes

1 The formula for calculating the I-CVI is: number of experts rating an item ≥ 3/the total number of experts [46, 47]. In this study, the S-CVI/Ave is used (mean value calculation method). The S-CVI/Ave is calculated by: Sum of I-CVI scores/number of items [46].
